# Supplementary figures and images for: Volumetric imaging of human mesenchymal stem cells (hMSCs) for non-destructive quantification of 3D cell culture growth
Source: PLoS One. 2023 Mar 28;18(3):e0282298. doi: 10.1371/journal.pone.0282298 (PMC10047548; doi:10.1371/journal.pone.0282298)

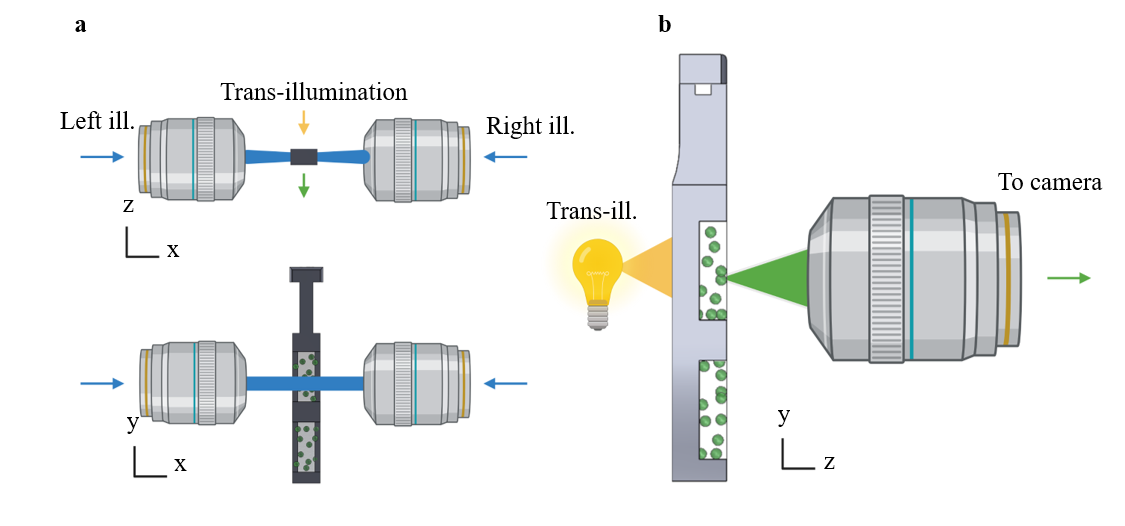

Supplement: S1 Fig — a) The chamber allows for dual-sided lightsheet and trans-illumination. b) The chamber can be scanned in all 3 dimensions and rotated for stitching and precise sample positioning for 3D optical imaging. Not to scale. (TIF) [file pone.0282298.s001.tif]

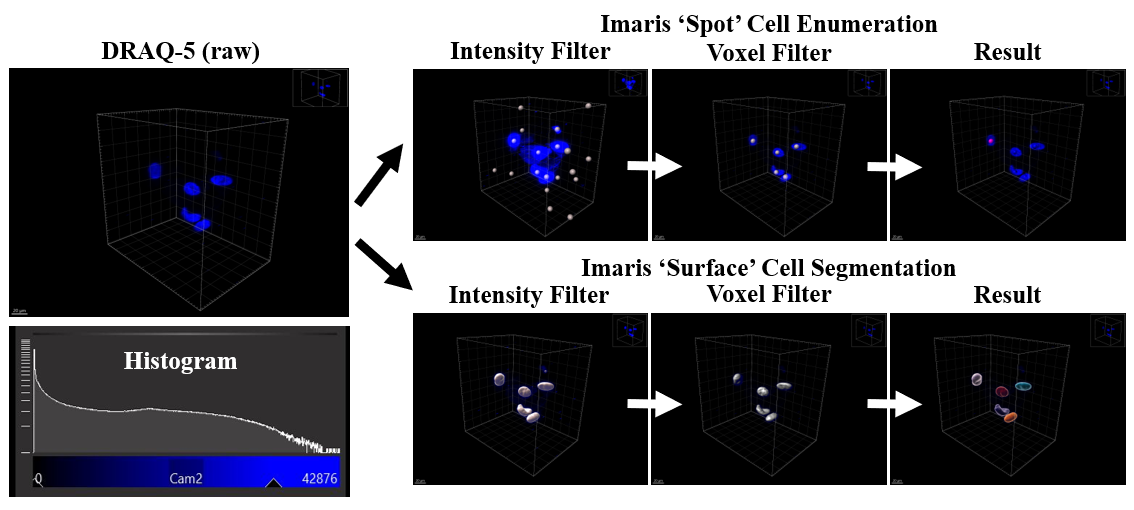

Supplement: S2 Fig — The segmented cell nuclei can be analyzed and classified by, for example, average distance to 3 nearest neighbors. (TIF) [file pone.0282298.s002.tif]

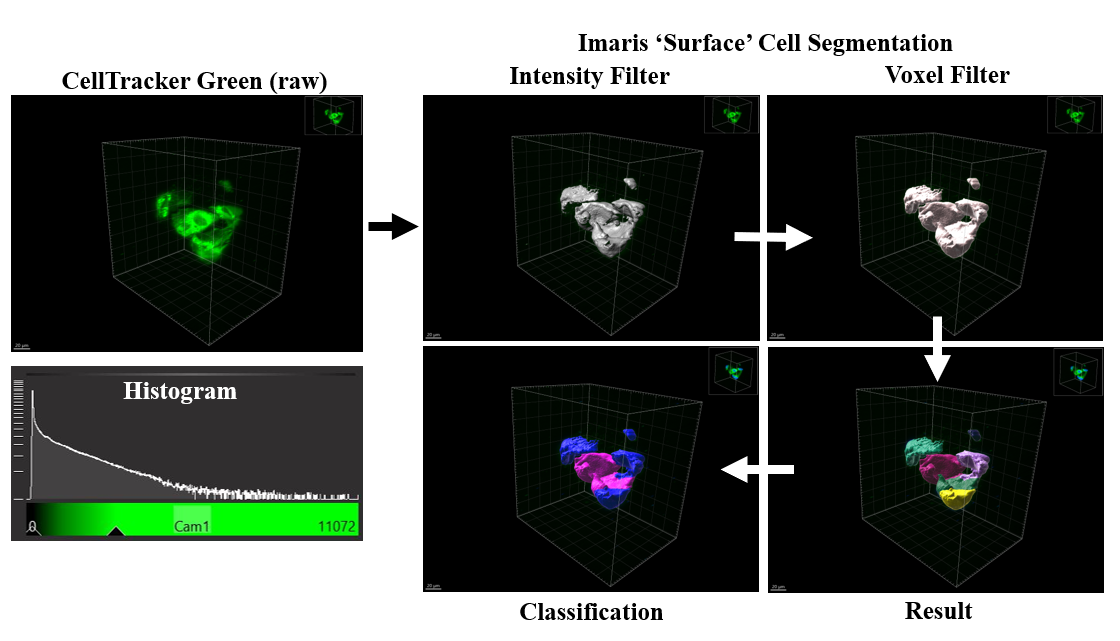

Supplement: S3 Fig — Single cells can be classified by volume using Imaris’ morphological-based segmentation. (TIF) [file pone.0282298.s003.tif]

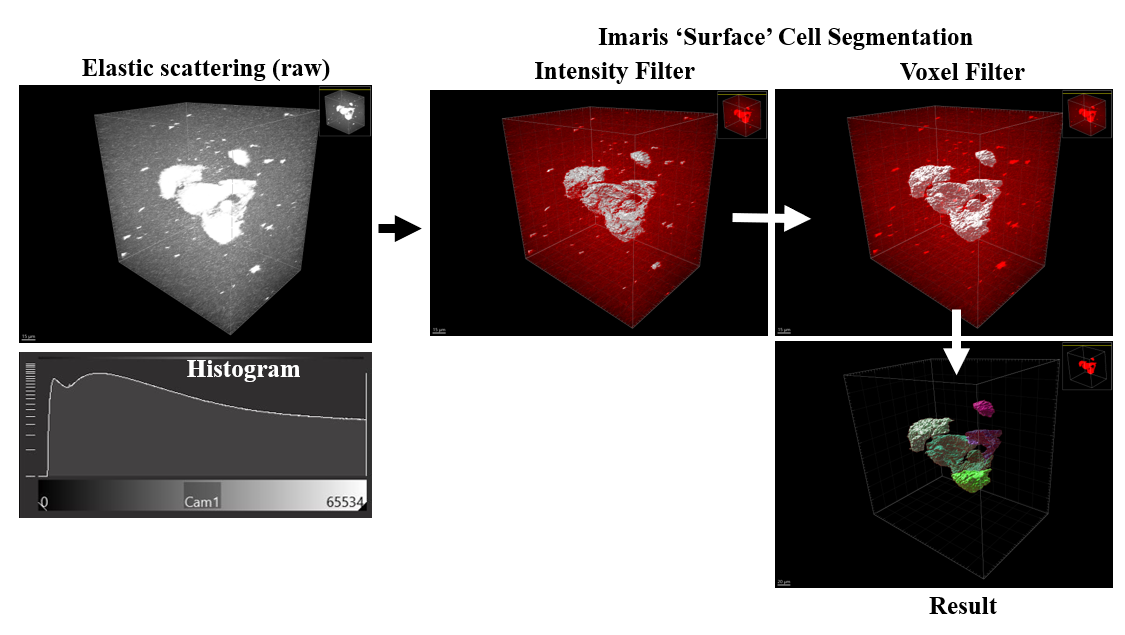

Supplement: S4 Fig — Cells scatter at higher intensity values than the microcarrier and surrounding agarose. After the high-pass intensity filter, the voxel filter removes small scatterers or debris in the agarose. (TIF) [file pone.0282298.s004.tif]

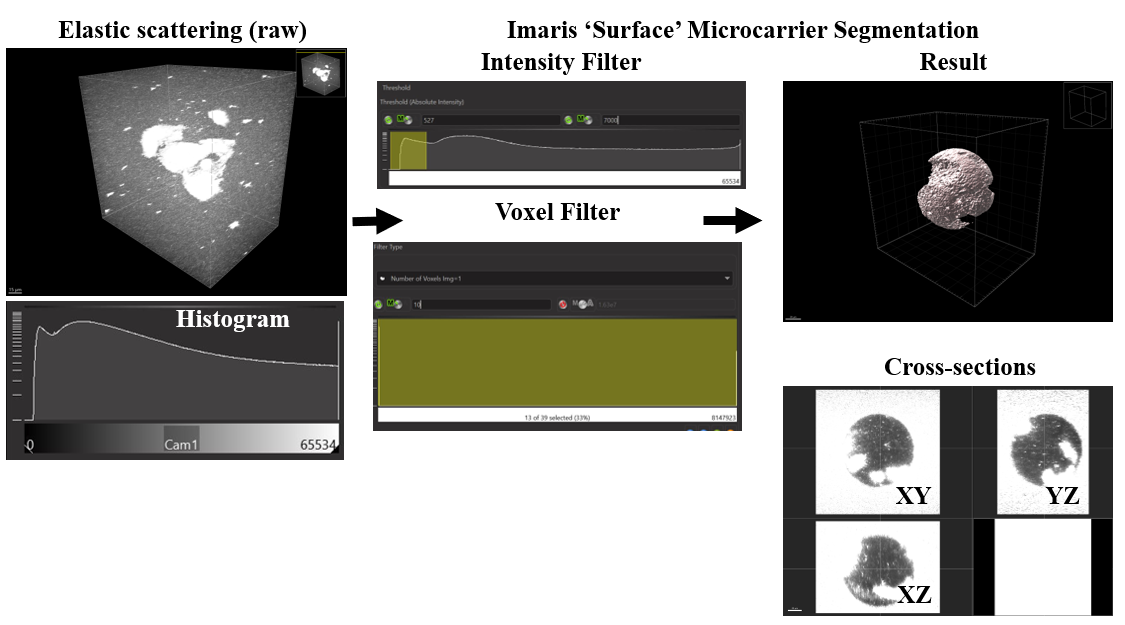

Supplement: S5 Fig — The hydrogel microcarriers scatter less than the cells and surrounding agarose. A low-pass intensity and 90 μm size filter identify individual spherical microcarriers. The en face and orthogonal cross-sections permit visualization of the gelMA microcarriers using elastic scattering contrast. (TIF) [file pone.0282298.s005.tif]

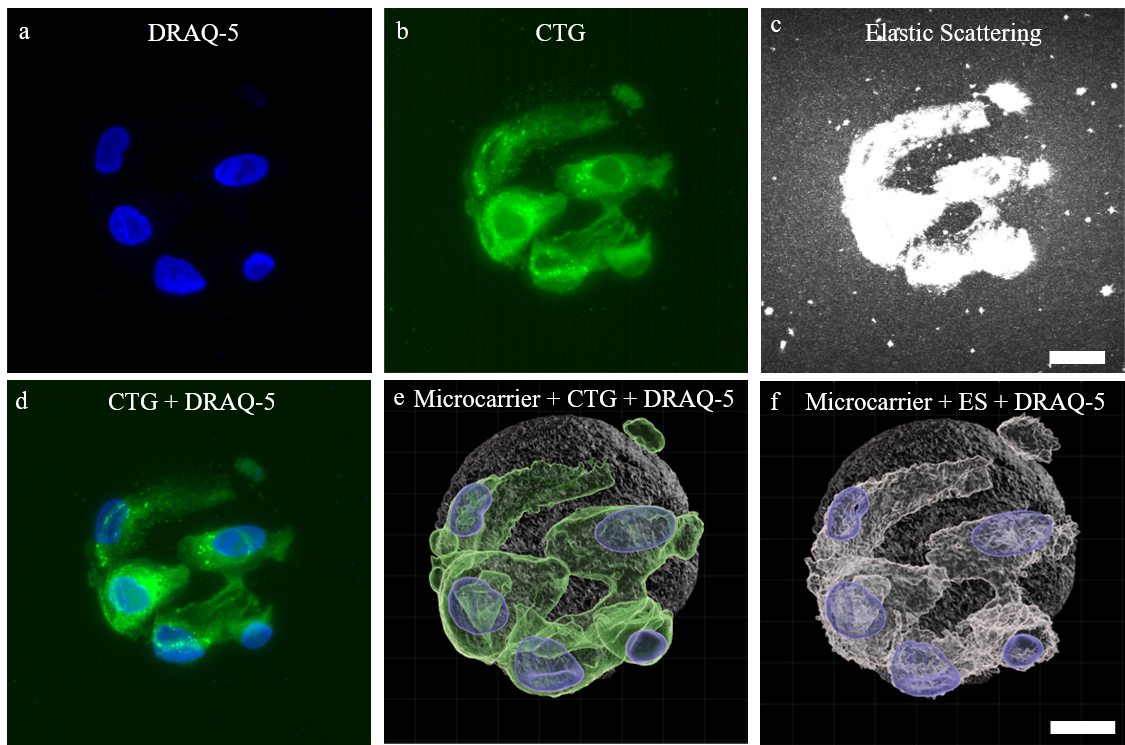

Supplement: S6 Fig — Max intensity projections of a passage 7 day 7 microcarrier using the raw a) DRAQ-5, b) CellTracker Green, and c) elastic scattering data. d) Merge of CTG + DRAQ-5 fluorescence projections. e) Merge of the segmented microcarrier, CTG (shaded green), and DRAQ-5 (shaded blue) cell regions. f) Merge of the segmented microcarrier, elastic scattering (shaded white), and DRAQ-5 cell volumes. Scale bar = 25 μm. (TIF) [file pone.0282298.s006.tif]
