# Supplementary material for: Volumetric imaging of human mesenchymal stem cells (hMSCs) for non-destructive quantification of 3D cell culture growth
Source: PLoS One. 2023 Mar 28;18(3):e0282298. doi: 10.1371/journal.pone.0282298 (PMC10047548; doi:10.1371/journal.pone.0282298)
Supplement: Attachment — Submitted filename: Review comments.pdf [file pone.0282298.s016.pdf]

This manuscript is about employing elastic scattering and fluorescence light sheet microscopy to quantify cell volume and cell number. It appears that the authors are demonstrating two techniques in this manuscript –

a) off-line fluorescence-based direct cell enumeration and cell volume quantification method
b) ELIAS method non-destructively monitors microcarrier-bioreactor cell culture growth without the need for cellular detachment,

While these two techniques complement each other, for clarity, the authors should consider separating into two standalone articles. Fluorescence-based imaging could be supporting data for the ELIAS. Thoughtful experiments are validated with sufficient results. This work can be published in PLOS One Journal after a minor revision.

- Figure 1 shows the fluorescence and elastic scattering images. The authors mention that the elastic scattering from cytoplasm is already reported, it is motioned that the new demonstration is elastic scattering from the nucleus.

- Vertical and horizontal scales of 1(m) are hard to read and hence conclude anything.

- Is there a limit to the cell volume size that this detection method can be applied?

- It is mentioned that method is semi-automated – but it is not clear the "automated" part.

- What is the imaging depth of this study? What is the dynamic range - the minimum and maximum cells/microcarrier – that the authors observed in this study. These two points were addressed in the manuscript, but the numbers are for ELIAS and not for this study. Also, instead of spelling out each and every advantage of ELIAS, perhaps authors should consider citing the advantages of the techniques in one or two sentences.
